# Biscogniauxone, a New Isopyrrolonaphthoquinone Compound from the Fungus *Biscogniauxia mediterranea* Isolated from Deep-Sea Sediments †

**DOI:** 10.3390/md14110204

**Published:** 2016-11-02

**Authors:** Bin Wu, Jutta Wiese, Rolf Schmaljohann, Johannes F. Imhoff

**Affiliations:** 1 GEOMAR Helmholtz Center for Ocean Research Kiel, RD3 Marine Microbiology, Düsternbrooker Weg 20, 24105 Kiel, Germany; wubin@zju.edu.cn (B.W.); jwiese@geomar.de (J.W.); rschmaljohann@geomar.de (R.S.); 2 Ocean College, Zhejiang University, Hangzhou 310058, China

**Keywords:** marine fungi, glycogen synthase kinase, acetylcholinesterase, *Biscogniauxia mediterranea*, deep sea

## Abstract

The properties and the production of new metabolites from the fungal strain LF657 isolated from the Herodotes Deep (2800 m depth) in the Mediterranean Sea are reported in this study. The new isolate was identified as *Biscogniauxia mediterranea* based on ITS1-5.8S-ITS2 and 28S rRNA gene sequences. A new isopyrrolonaphthoquinone with inhibitory activity against glycogen synthase kinase (GSK-3β) was isolated from this fungus. This is the first report of this class of compounds from a fungus isolated from a deep-sea sediment, as well as from a *Biscogniauxia* species.

## 1. Introduction

An enormous number of natural products have been isolated and identified from marine microbiota, but deep-sea marine microorganisms remain an almost untapped resource [1]. As methods for sample collection, identification, and culturing technologies of deep-sea microorganisms have advanced significantly in recent years, a shift of foci from shallow water to the deep sea for natural product chemists has come along [2]. During studies on the diversity of fungi in the deep Mediterranean Sea and also on sponges, in particular in *Tethya aurantium* [3], a valuable tool for the identification of fungal isolates proved to be the combination of morphological criteria and the comparison of the ITS1-5.8S-ITS2 fragment sequences [3,4]. In particular, the sequence information gave valuable additional information for the phylogenetic evaluation of the investigated isolates. Our studies with sediments from the deep Mediterranean Sea have shown the dominance of representatives of *Aspergillus* and *Penicillium* (almost half of all isolates) together with isolates from 10 other genera, including strain LF657 as a single isolate (out of a total of 43) of *Biscogniauxia* [4]. This *Biscogniauxia* isolate was obtained from sediments of the Herodotes Deep (2800 m depth) in the Mediterranean Sea and properties of this fungus and the production of metabolites are reported in the present study. A new isopyrrolonaphthoquinone was identified in extracts of the *Biscogniauxia* isolate together with a known isocoumarin and a known cyclopentapeptide. As reviewed by Piggott (2005), the natural product backbones of isofuranonaphthoquinones and related compounds comprise a small group of secondary metabolites isolated from fungi, plants, bacteria, and insects, and these compounds have a broad spectrum of biological effects, such as antibiotic, antioxidant, or antiplasmodial activities as well as cytotoxicity towards tumor cell lines and inhibition of the myosin light-chain kinase [5]. Here we report the first discovery of an isopyrrolonaphthoquinone compound from a fungus isolated from deep-sea sediment.

## 2. Results

### 2.1. Sampling, Isolation, and Identification of Strain LF657

**Figure 1:**
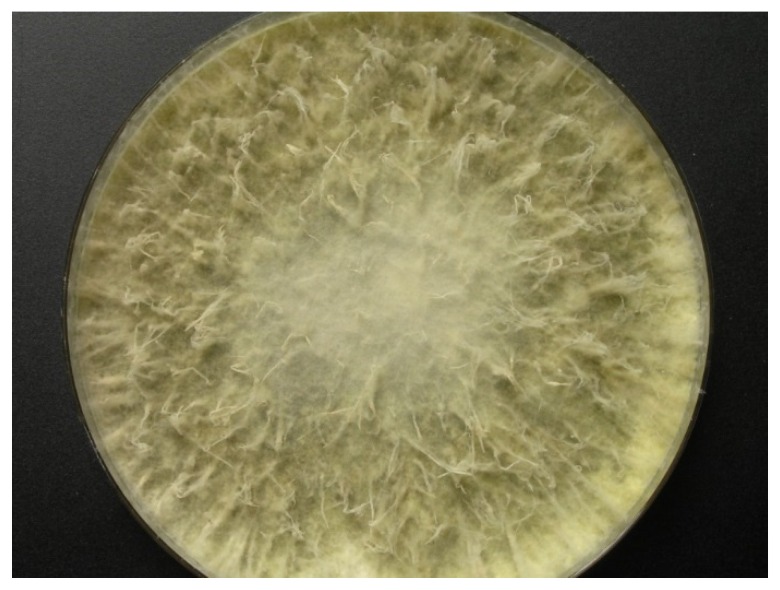
Colony of *Biscogniauxia mediterranea* strain LF657 grown on Wickerham agar (26 °C, 10 days).

During the Meteor 71/2 cruise to the Eastern Mediterranean Sea, samples were collected from the sediment of the Herodotes Basin (2800 m water depth), diluted in sterile Mediterranean Sea water, and plated onto five different media with the aim to isolate bacteria and fungi. Strain LF657 was obtained from a sample taken from the top sediment layer (0 to 0.5 cm depth) after cultivation on a low-nutrient “Cytophaga-Flavobacterium-Bacteroides” (CFB) agar. The isolate grew well on Wickerham agar medium containing 3% sodium chloride and also on glucose-peptone-yeast extract agar supplemented with sea water with an approximately 3.5% salt concentration with a colony diameter of 100 mm after 10 days of incubation at 26 °C (Figure 1). No reproductive structures were observed, which would enable the identification on a morphological basis.

The fungus was identified by its ITS1-5.8S-ITS2 gene fragment sequence (506 nucleotides), which was 99.1% identical to several *Biscogniauxia mediterranea* strains, such as *B. mediterranea* G19 (acc. No. KT364486), *B. mediterranea* CCTU125 (acc. No. KX267752), and *B. mediterranea* BM07.003 (acc. No. FR734187). Also the 28S rRNA gene sequence (837 nucleotides) revealed members of *Biscogniauxia mediterranea* as the next related strains. Among them were *B. mediterranea* Bm12.022 (acc. No. KM216771) with a similarity of 100% and *B. mediterranea* AS (acc. No. KR909208) with 99.5% similarity. Therefore, strain LF657 was classified as *Biscogniauxia mediterranea* (Sordariomycetes, Xylariales, Xylariaceae). The sequences of the ITS1-5.8S rRNA-ITS2 gene fragment and of the 28S rRNA gene fragment from LF657 were submitted to the Genbank database and were assigned to accession numbers KX982261 and KX982262, respectively.

*Biscogniauxia* was first described by Kuntze [6] and is a genus assigned to the family Xylariaceae. *Biscogniauxia* species are known plant pathogens, which typically are bark parasites and are able to degrade major components of the wood, cellulose and lignin. Though *Biscogniauxia mediterranea* became known as the causative agent of charcoal canker in Mediterranean cork oak, it appears not to be host specific and apparently is distributed worldwide [7,8]. There is no evidence so far on the occurrence of *Biscogniauxia* species in marine waters or even in the deep sea. A recent study using 5.8S rRNA gene and ITS2 sequences with a larger number of isolates has given interesting insight into the phylogeny of the Xylariaceae family [7]. With some exceptions, this phylogenetic analysis showed a reasonable degree of correlation with the classification based upon morphological properties. A reevaluation of the phylogenetic relationship of the Xylariaceae was performed on the basis of sequences of several genes, including those coding for ß-tubulin, the second largest subunit of RNA polymerase II (RPB2), and the large subunit ribosomal RNA (LSU rDNA). This study revealed a separate common lineage of *Biscogniauxia* and *Camillea* species, which are usually considered to be very closely related genera if not synonyms, and clearly indicated their separation from other clades of this family [8]. The position of *Biscogniauxia* in both studies was fully consistent with the morphological and chemotaxonomic characters that assimilate this genus to the Xylariaceae.

### 2.2. Structural Elucidation

**Figure 2:**
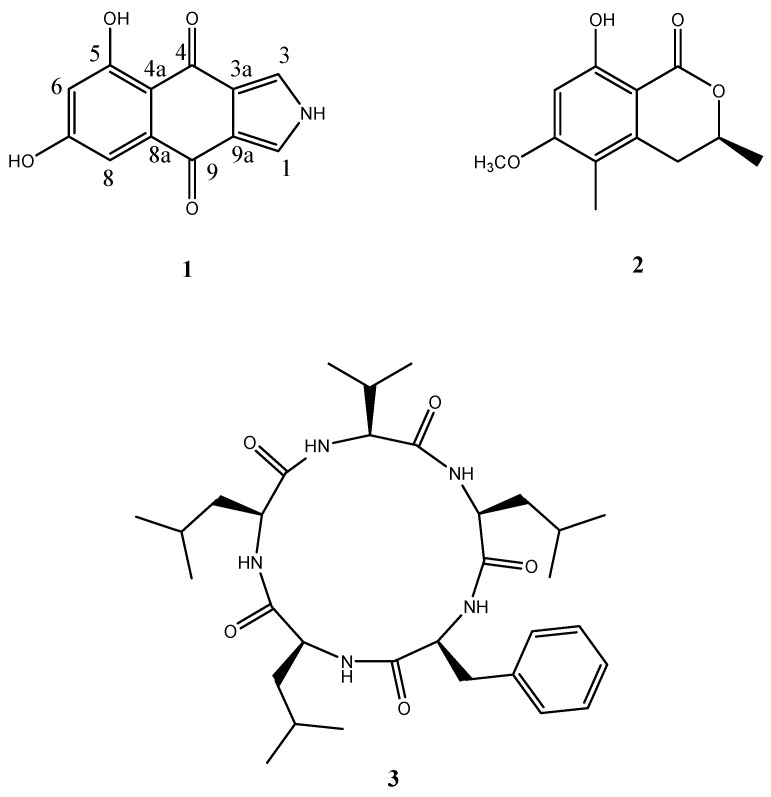
The structures of the compounds identified from *Biscogniauxia mediterranea* strain LF657.

The MeOH extracts of the culture broth and the mycelia of strain LF657 were subjected to HPLC-DAD(UV)-MS analysis, revealing more than 10 compounds. Applying preparative HPLC (C_18_), seven compounds were isolated. Among them, a new isopyrrolonaphthoquinone **1**, together with a known isocoumarin, 6-methoxy-5-methyl mellein (**2**) [9], and a cyclopeptide, cyclo-(l-Phe-l-Leu-l-Val-l-Leu-l-Leu) (**3**), a previously synthesized derivative of the fungal metabolite sansalvamide A, were identified [10,11] (Figure 2). Those three compounds were detected in both the mycelium and the culture broth, but the yields were higher in the mycelium extract. The production of derivatives of compound **1** by strain LF657 is expected because of the presence of metabolites with similar UV signals in the extracts.

**Figure 3:**
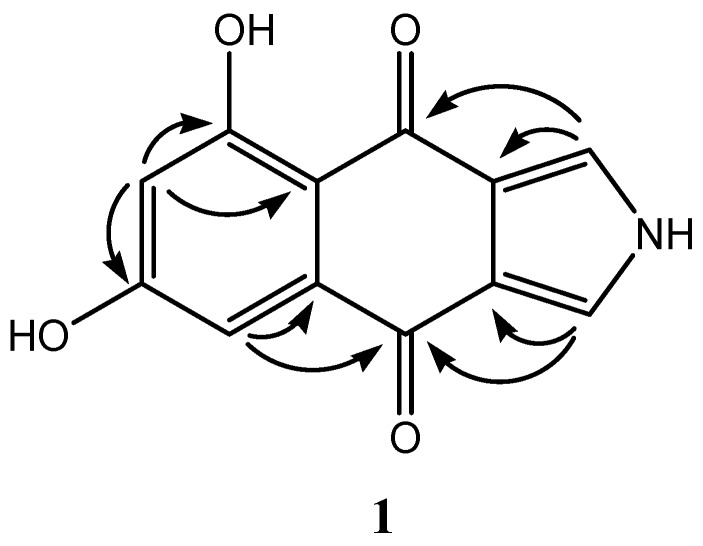
Key HMBC correlations of compound **1**.

Compound **1** was isolated as a brown powder. The molecular formula was determined to be C_12_H_7_NO_4_ by analysis of the HR-TOF-MS ion peak at *m/z* 252.0277 [M + Na]^+^ (calcd. 252.0267). The ^1^H and ^13^C NMR spectra of **1** (Table 1) showed similar chemical shifts and the same multiplicities for 12 carbon atoms of rings A, B and C as compared to the known compound monosporascone with a minor difference, indicating that compound **1** supposedly possesses the basic structure of a naphtho[2,3-*c*]furandione (isofuranonaphthoquinone), a minor natural compound class. Both isobenzofuran and benzofuran substructures exist in nature, with the benzofuran found in the majority of structures. The characteristic proton signals at δ_H_ 7.70 (overlap, dd, *J* = 3.1, 1.6 Hz) for two protons in the furan rings in the ^1^H NMR spectrum of **1** and the HMBC cross-peaks from this overlapped proton signals to both carbonyl carbons at δ_C_ 179.1 and 185.2 were observed. This indicated the presence of the isobenzofuran-4,7-dione instead of a benzofuran. Two main subclasses exist among the hitherto known naphtho[2,3-*c*]furandinone natural products. One includes naphtho[2,3-*c*]furan-4(9*H*)-ones with two carbonyls at the 5 and 8 positions, which often undergo a tautomeric relationship with naphtho[2,3-*c*]furan-4-ols [5]. Another contains naphtho[2,3-*c*]furan-4,9-diones with two carbonyls at the 4 and 9 positions. To clarify the position of the two carbonyls in the supposed isofuranonaphthoquinone ring system and the substitution pattern, detailed 2D NMR experiments were carried out. The long-range correlations from the aromatic proton at δ_H_ 7.06 (d, *J* = 2.4 Hz, H-8) and the isobenzofuran proton at δ_H_ 7.70 to the same carbonyl carbon at δ_C_ 179.1 (C-9) suggested the presence of a naphtho[2,3-*c*]furan-4,9-dione backbone. Two aromatic proton signals at δ_H_ 6.47 and 7.06 were deduced to be *meta* to each other from the analysis of their coupling constant value of 2.4 Hz and the HMBC cross-peaks of H-6/C-4a, H-6/C-5 and H-8/C-8a. The OH signal at δ_H_ 13.27 was positioned at C-5 since it showed a sharp singlet which derived from the intramolecular hydrogen bond with the remaining C=O at δ_C_ 185.2. Thus, another hydroxyl must locate at C-7 which was also confirmed by the HMBC cross-peaks of H-6,8/C-7. Although the ^1^H NMR data of **1** exhibited good similarity to a number of isofuranonaphthoquinone compounds [12], two supposedly furan CH resonances at C-1 and C-3 were observed to upshift approximately 23 ppm, which implied a NH instead of an O linkage between C-1 and C-3. This inference also was supported by the HR-TOF-MS results and indicates the presence of an N atom in the molecule of **1**. Only few known compounds possess the *N*-containing isopyrrolonaphthoquinone structures, such as azamonosporascone [12], bhimamycin C and D [13] as well as bhimamycin F, H, and I [14]. According to the established data (Figure 3, Table 1), compound **1** was identified as a new N-atom-containing isopyrrolonaphthoquinone compound named biscogniauxone. As furanes are easily transformed into pyrroles by a reaction with primary amines [13], the isopyrrolonaphthoquinone may derive from its isofuranonaphthoquinone precursor. The ^1^H and ^13^C NMR spectra of compound **1** including COSY, HMQC, HMBC and HRESIMS are given in the supplementary material (Figures S1–S7), the complete NMR signal assignments are listed in Table 1.

### 2.3. Biological Activities

The most important activities observed for the new compound **1** were inhibiting glycogen synthase kinase (GSK-3β) with an IC_50_ value of 8.04 μM (±0.28) and the known cyclopentapeptide (**3**) showing an inhibitory effect on acetylcholinesterase (AchE) by a minimal inhibition concentration (MIC) of 5.87 μM (±0.74). Positive controls were 4-Benzyl-2-methyl-1,2,4-thiadiazolidine-3,5-dione (TDZD-8) for GSK-3β, which revealed an IC_50_ value of 0.26 ± 0.03 μM, and huperzine for AchE with an IC_50_ value of 0.012 μM (±0.0009). Compound **1** very weakly inhibited the growth of *Staphylococcus epidermidis* and methicillin-resistant *Staphylococcus aureus* with IC_50_ values in the range of 100 μM, respectively. *Septoria tritici* and *Trichophyton rubrum* were weakly inhibited by compound **2** in the same concentration range.

## 3. Discussion

In view of their parasitic-pathogenic nature, the production of bioactive compounds and, in particular, of phytotoxic substances has been studied in a number of *Biscogniauxia* strains, and several bioactive substances have been described for the first time from these fungi. A new phytotoxic biscopyran is produced by *Biscogniauxia mediterranea* together with phenylacetic acid and the isocoumarin 5-methylmellein [15]. Two new azaphilone derivatives, biscogniazaphilones A and B, with antimycobacterial activity were produced by *Biscogniauxia formosana* together with nine known compounds [16]. In addition, two new substances, biscognin A and B, were found in extracts of *B. mediterranea* [17]. The new guaiane sesquiterpene xylaranone and the known terpenoid xylaranol B (together with mellein derivatives) were isolated from *Biscogniauxia nummularia*. They revealed antigerminative activity against seeds of *Raphanus sativus* [18].

In extracts of *B. mediterranea* strain LF657, isolated from a deep-sea sediment of the Eastern Mediterranean Sea at 2800 m water depth, the new isopyrrolonaphthoquinone compound biscogniauxone (**1**) was identified and found to exhibit inhibitory activity against the enzyme GSK-3β, which is an important target for the treatment of diseases such as diabetes type 2, neurological disorders, and cancer [19,20,21].

In addition to biscogniauxone (**1**), two further metabolites were identified: a mellein derivative, identified as 6-methoxy-5-methyl mellein (**2**), and the cyclic pentapeptide cyclo-(l-Phe-l-Leu-l-Val-l-Leu-l-Leu, sansalvamide A amide (**3**). Compound **3** (C_32_H_51_N_5_O_5_) is structurally very similar to the cyclic pentadepsipeptide sansalvamide A (C_32_H_50_N_4_O_6_), which contains leucic acid (Oleu) instead of leucin in comparison to compound **3** [11]. Sansalvamide A was found in extracts of a *Fusarium* isolate obtained from the marine seagrass *Halodule wrightii.* Because of promising cytotoxic effects on several cancer cell lines, sansalvamide A as well as a sansalvamide A amide and derivatives of both compounds were synthesized [22]. It was shown that improved activities against the drug-resistant colon cancer cell line HCT-116, the interaction with the key oncogenic protein Hsp90, as well as the ADME (adsorption, distribution, metabolism, excretion) properties were obtained with some derivatives, especially with (R,R)betaOH(Bn)-Phe-Leu-N-Me-Val-d-Leu-d-Phe [22].

## 4. Experimental Section

### 4.1. General Experimental Procedures

^1^H NMR (500 MHz) and ^13^C NMR (125 MHz) spectra were measured at 25 °C on a Bruker (Bremen, Germany) AVANCE DMX 500 NMR spectrometer with tetramethylsilane (TMS) as internal standard. The signals of the residual solvent protons and the solvent carbons were used as internal references (δ_H_ 2.5 ppm and δ_C_ 39.5 ppm for DMSO-*d_6_* (compound **1**), δ_H_ 3.31 ppm and δ_C_ 49.0 ppm for methanol-*d*_4_ compound **2** and **3**). High-resolution mass spectra were acquired on a benchtop time-of-flight spectrometer (micrOTOF II, Bruker Daltonics, Bremen, Germany) with positive electrospray ionization (ESI).

### 4.2. Isolation, Cultivation, and Storage of the Producer Strain LF657

Strain LF657 was obtained from a sample collected during the cruise Meteor 71/2 in December 2006 to January 2007 to the Eastern Mediterranean Sea from the top sediment layer (0 to 0.5 cm depth) of the Herodotes Basin at 2800 m water depth [23]. The Herodotos Basin is a large basin in the most oligotrophic eastern part of the Mediterranean Sea extending southwestern from Cyprus Island. Sediment samples from one of the sampling sites (33°42.989 N, 26°20.329 E) were taken with a multi corer, subsampled aseptically, diluted 10^−1^ to 10^−4^ in sterile Mediterranean Sea water and spread onto five different agar media [23]. Strain LF657 was isolated from a 10^−1^ dilution plated onto Cytophaga-Flavobacterium-Bacteroides medium (0.1% BBL™ (Becton, Dickinson and Company, Franklin Lakes, NJ, USA) tryptone, 0.05% Bacto™ (Becton, Dickinson and Company, Franklin Lakes, NJ, USA), yeast extract, 0.05% CaCl_2_·2H_2_O, 0.05% MgCl_2_·7H_2_O, and 1.5% Bacto™ agar dissolved in Mediterranean Sea water, pH = 7.0). Sub-cultivation was performed using glucose-peptone-yeast extract agar medium (GPY; 0.1% glucose, 0.05 peptone, 0.01% yeast extract, and 1.5% Bacto™ agar dissolved in sea water; pH = 7.2–7.4) and a modified Wickerham-medium (WSP30; 1% glucose, 0.5% peptone, 0.3% yeast extract, 0.3% malt extract, 3% sodium chloride, and 1.5% Bacto™ agar; pH = 6.8) [24]. The isolate is stored in liquid nitrogen and in addition at −80 °C using the CRYOBANK™system (MAST Diagostika GmbH, Reinfeld, Germany).

### 4.3. Identification of the Strain LF657

The genetic characterization of the fungus was performed by the analysis of the ITS and 28S rRNA gene sequences. DNA-extraction, amplification of the ITS1-5.8S rRNA-ITS2 fragment, and sequencing procedure were carried out according to Wiese et al. [3]. Amplification of the 28S rRNA gene fragment was processed as described by Wu et al. [25]. Briefly, the fungal cells were raptured by homogenization by using glass beads and the Precellys 24 system (Bertin Technologies, Montigny-le-Bretonneux, France). The suspension was centrifuged at 6000× *g* for 10 min and the supernatant was stored at −20 °C. This DNA extract was used as template for amplification of the desired gene fragments applying the following primers: ITS1 (5′-TCCGTAGGTGAACCTGCGG-3′) and ITS4 (5′-TCCTCCGCTTATTGATATGC-3′) for the ITS1-5.8S rRNA-ITS2 fragment as well as 5.8SR (5′-TCGATGAAGAACGCAGCG-3′) and LR7 (5′-TACTACCACCAAGATCT-3′) for the 28S rRNA gene fragment, respectively [26,27]. Sequencing of the amplicons was performed with the primers ITS1 and ITS4 as well as with LR0R (5′-ACCCGCTGAACTTAAGC-3′) and LR5 (5′-TCCTGAGGGAAACTTCG-3′), respectively [28]. Closest relatives were identified by sequence comparison with the NCBI Genbank database using BLAST (Basic Local Alignment Search Tool) [29]. Sequence similarity values were determined with the “bl2seq” tool of the NCBI database [30].

### 4.4. Fermentation and Production of Extracts for the Purification of Compound ***1***, ***2***, ***3***

Strain LF657 was inoculated onto agar plates containing WSP30 medium. After incubation for 13 days at 26 °C the pre-culture was used as inoculum of Erlenmeyer flasks containing 750 mL SM medium (2% soy peptone, 2% mannitol, 1.5% Tropic Marine Salt, pH 7.0). The flasks were incubated for 16 days at 28 °C as static cultures in the dark. The mycelium was separated from the culture broth. The 4.5 L fermentation broth was extracted using ethyl acetate (2.4 L). The upper phase was evaporated to dryness. The residue was dissolved in 5 mL methanol to obtain the extract of the culture broth. The mycelium was extracted by adding 0.9 L of ethanol and homogenizing using an ultra-turrax. After centrifugation at 10,000 rpm for 10 min, the supernatant was collected and evaporated to dryness. The resulting residue was dissolved in 5 mL methanol to get the extract of the mycelium. Crude extracts were stored at 4 °C until further use.

### 4.5. Extraction and Isolation of Compounds ***1***

Analytical reversed phase HPLC-DAD(UV)-MS experiments were performed using a C_18_ column (Phenomenex (Torrance, CA, USA) Onyx Monolithic C18, 100 × 3.00 mm) applying an H_2_O/acetonitrile (ACN) gradient with 0.1% formic acid added to both solvents (gradient: 0 min 5% ACN, 4 min 60% ACN, 6 min 100% ACN; flow 2 mL/min each; 6.1 min 100% ACN, 6.8 min 100% ACN, 7.0 min 5% ACN, 8.2 min 5%; flow 2.5 mL/min each) on a VWR Hitachi (Darmstadt, Germany) Elite LaChrom system with an L-2450 diode array detector, an L-2130 pump, and an L-2200 autosampler (VWR, Darmstadt, Germany). The system was coupled to an ESI-ion trap detector with positive ionization (Esquire 4000, Bruker Daltonics, Bremen, Germany) for mass detection.

The preparative HPLC was conducted with a HPLC-UV system (LaPrep, pump P110, UV detector P311, smartline 3900 autosampler (VWR International, Darmstadt, Germany)) coupled with a LABOCOL Vario-2000 fraction collector (LABOMATIC, Weil am Rhein, Germany) using a C_18_ column (Phenomenex Gemini-NX C18 110A, 100 mm × 50mm). Purification of compound **1** was performed with a gradient from 10% (0 min) to 15% (30 min) acetonitrile (ACN) and a flow of 15 mL/min yielding 10.0 mg (*t*_R_ 9.1 min). Compound **2** and **3** were purified applying the following gradient parameters: 0 min 10% ACN, 17.5 min 60% ACN, 22 min 100% ACN at a flow of 100 mL/min. The yield of **2** was 32.1 mg (*t*_R_ 16.8 min) and of **3** 213.9 mg (*t*_R_ 19.8 min).

Biscogniauxone (**1**) had the following properties: white powder; UV (MeOH) λ_max_ (log ε) 254 (4.17), 396 (3.80) nm; ^1^H NMR and ^13^C NMR, see Table 1; ESIMS *m*/*z* 252 [M + Na]^+^; HR-TOF-MS *m*/z 252.0277 [M + Na]^+^ (calcd. for C_12_H_7_NO_4_Na, 252.0267).

### 4.6. Biological Activities Assays

The antimicrobial activities of compounds **1** and **2** against the bacteria *Bacillus subtilis* as well as the human pathogenic yeast *Candida albicans* (DSM 1386) were determined according to Ohlendorf et al. [31]. The bioassays with the clinically relevant bacterial strains *Staphylococcus epidermidis* (DSM 20044), methicillin-resistant *Staphylococcus aureus* (MRSA) (DSM 18827), and the causative agent of acne, *Propionibacterium acnes* (DSM 1897^T^), were performed as described by Silber et al. [32]. The phytopathogenic fungus *Septoria tritici* and the dermatophytic fungus *Trichophyton rubrum* as well as the cytotoxic effect on the human hepatocellular liver carcinoma cell line HepG2 were tested according to Jansen et al. [33]. Inhibition of the enzymes glycogen synthase kinase (GSK-3β), acetylcholinesterase (AchE), phosphodiesterase (PDE-4B2), and protein tyrosine phosphatase (PTP1B) was determined according to Baki et al. [34], Ohlendorf et al. [31], Schulz et al. [35], and Silber et al. [36], respectively. The concentration of the compounds used in the initial bioassays was 100 μM (antibiotic tests), 50 μM (cytotoxic tests), and 10 μM (enzymatic tests). To determine the IC_50_ values concentrations of the compounds ranging from 0.1 μM to 50 μM were analyzed twice in duplicates.

## 5. Conclusions

For the first time, *Biscogniauxia mediterranea,* known as a plant parasitic-pathogenic fungus, was isolated from marine deep-sea sediments. In extracts of *B. mediterranea* strain LF657, isolated from the Eastern Mediterranean Sea at 2800 m water depth, one of the compounds was identified as the new isopyrrolonaphthoquinone, biscogniauxone (**1**), a new member of a minor natural compound class, and found to exhibit inhibitory activity against the enzyme GSK-3β with an IC_50_ value of 8 μM. This effect is much lower in comparison to the GSK inhibitor TDZD-8. However, it is of interest that another isopyrrolonaphthoquinone, namely bhimamycin H, also inhibited the activity of this enzyme in a similar range (IC_50_ value of 18 μM) [14]. Therefore, isopyrrolonaphthoquinones and similar structures can be considered as possible candidates for the development of drugs in order to treat diseases related to the biological target GSK-3β, such as diabetes type 2, neurological disorders, or cancer [19,20,21]. These findings are a nice example to demonstrate anew that natural products from the microbial treasure box of the ocean and derivatives thereof can be of great benefit for human health.

## Figures and Tables

**Table 1:** NMR spectroscopic data (500 MHz, DMSO-*d*_6_) of compound **1**.

Position	δ_C_^a,b^	δ_H_^c^ Mult. (*J* in Hz)	HMBC ^d^
1	123.9, CH	7.70, overlap, dd (3.1, 1.6)	3, 9a, 9
3	124.4, C		
3a	121.6 ^e^, CH	7.70, overlap, dd (3.1, 1.6)	1, 3a, 4, 9a
4	185.2, C		
4a	110.8, C		
5	165.4, C		
6	107.3, CH	6.47, d (2.4)	4a, 5
7	165.4, C		
8	108.4, CH	7.06, d (2.4)	7, 8a, 9
8a	138.2, C		
9	179.1, C		
9a	122.0 ^e^, C		
5-OH		13.27, s	

^a^ Recorded at 125 MHz; ^b^ Multiplicities inferred from DEPT and HMQC experiments; ^c^ Recorded at 500 MHz; ^d^ Proton showing long-range correlation with indicated carbons; ^e^ interchangeable.

## Supplementary Materials

NMR data of compound **1** are available online at www.mdpi.com/1660-3397/14/11/204/s1 in Figures S1–S7.
