# Supplementary material for: Biscogniauxone, a New Isopyrrolonaphthoquinone Compound from the Fungus Biscogniauxia mediterranea Isolated from Deep-Sea Sediments
Source: Mar Drugs. 2016 Nov 2;14(11):204. doi: 10.3390/md14110204 (PMC5128747; doi:10.3390/md14110204)
Supplement: Supplementary file 1 [file marinedrugs-14-00204-s001.pdf]

Supplementary Materials: Biscogniauxone, A New Isopyrrolonaphthoquinone Compound from the Fungus *Biscogniauxia mediterranea* Isolated from Deep-Sea Sediments

Bin Wu, Jutta Wiese, Rolf Schmaljohann and Johannes F. Imhoff

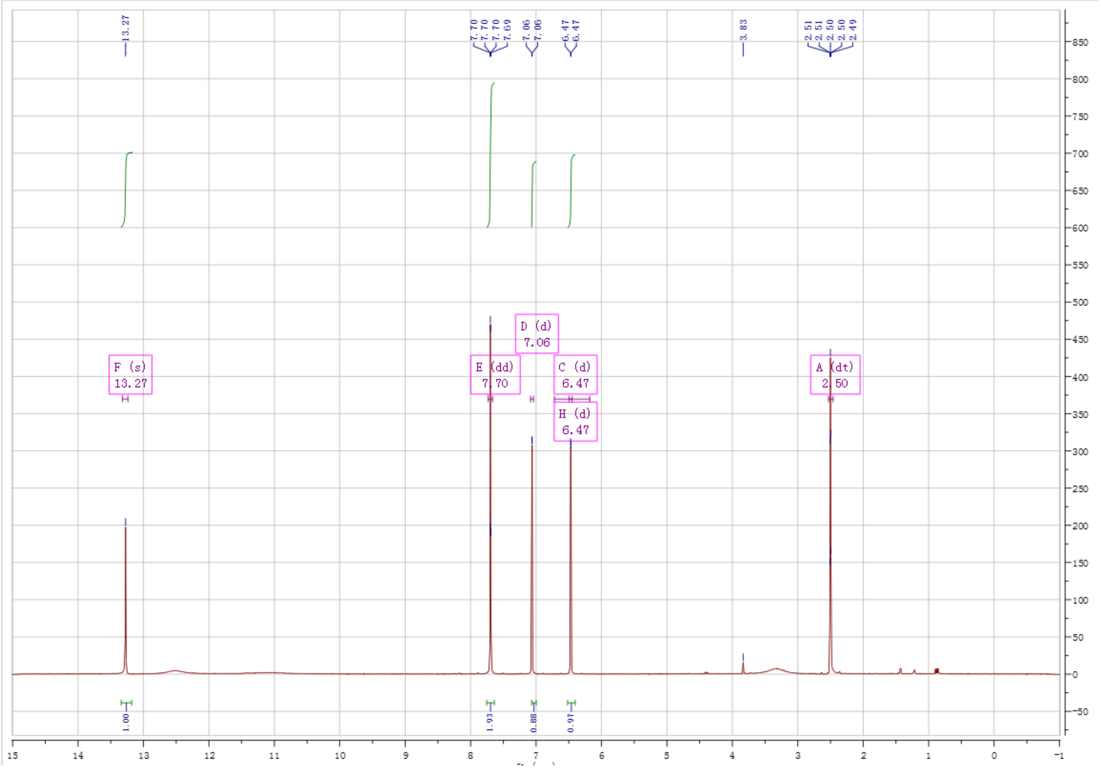

Figure S1. ^{1}H NMR in DMSO-d_6 for compound **1**.

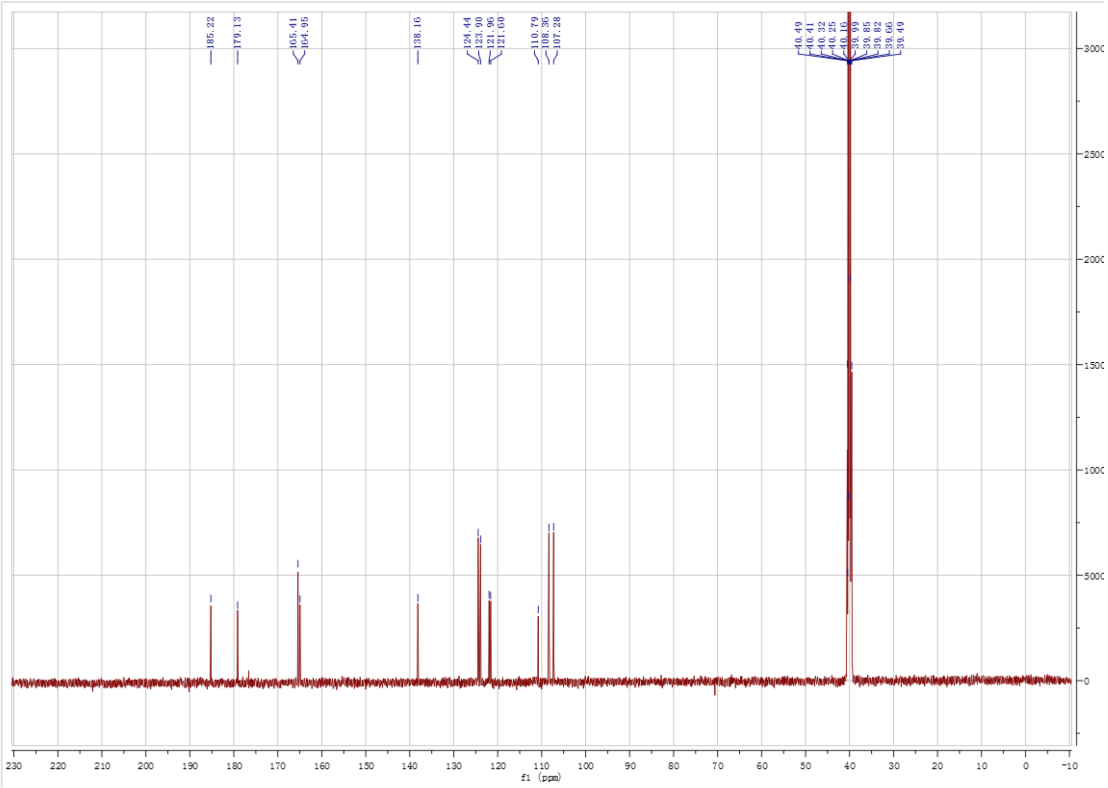

Figure S2. ^{13}C NMR in DMSO-d_6 for compound **1**.

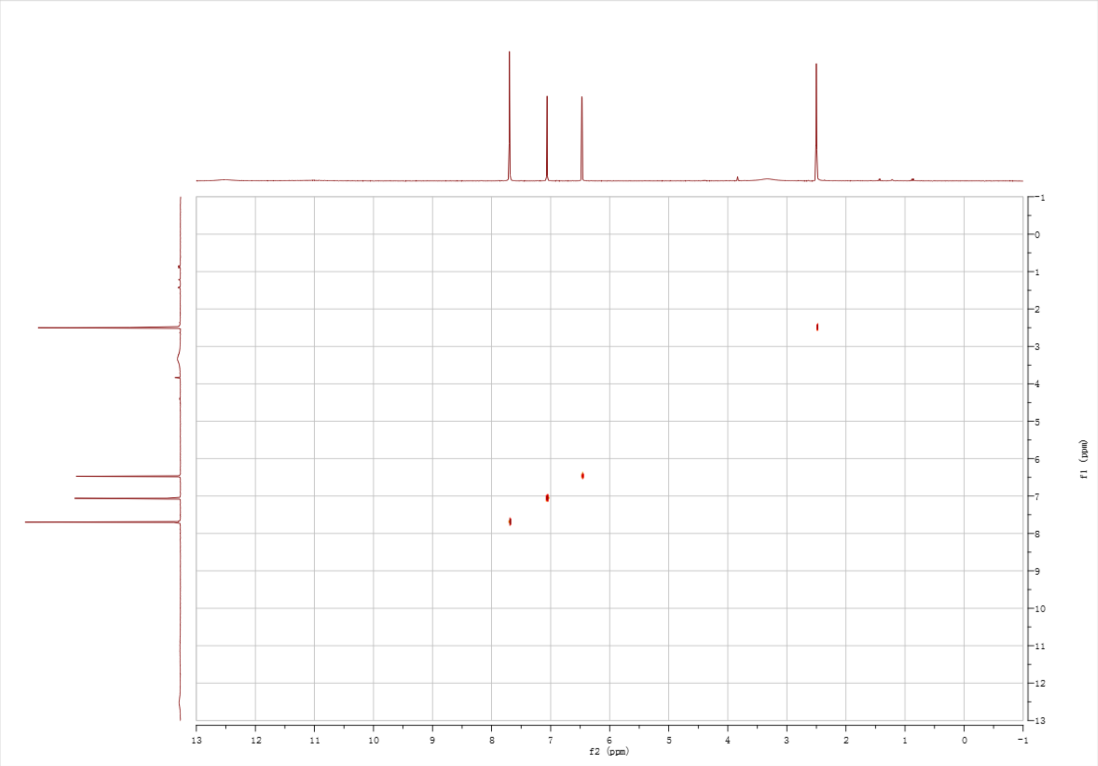

Figure S3. COSY in DMSO-d_6 for compound **1**.

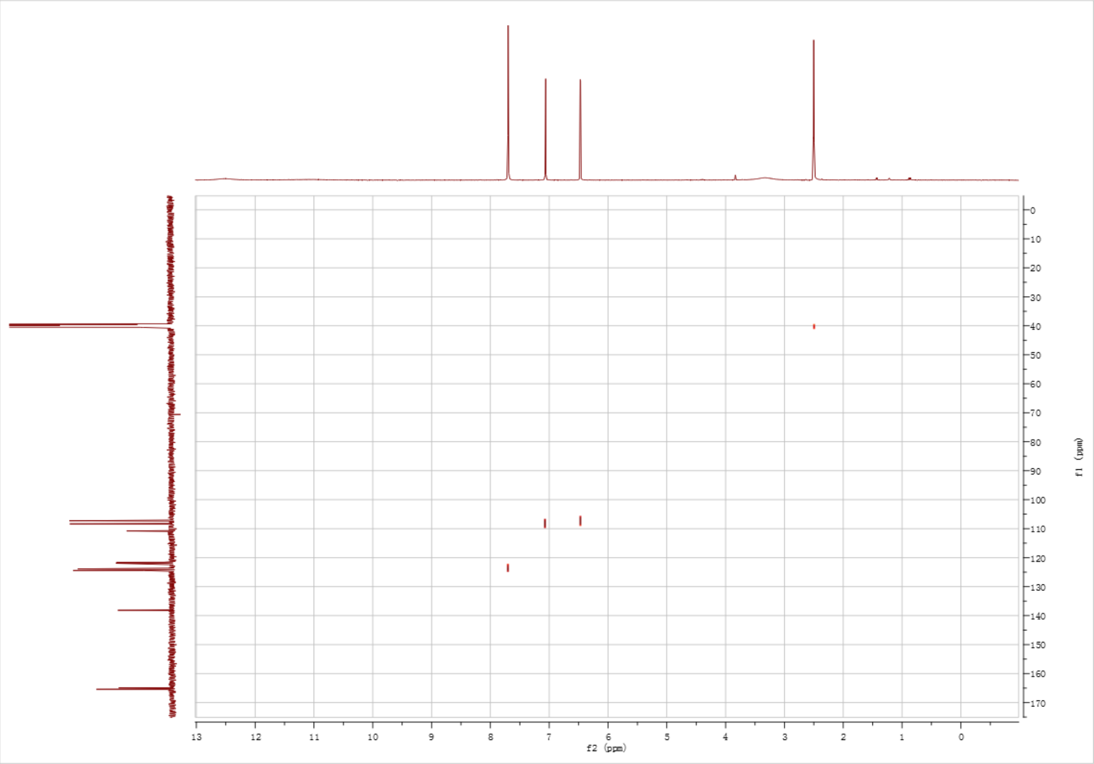

Figure S4. HMQC in DMSO-*d*6 for compound **1**.

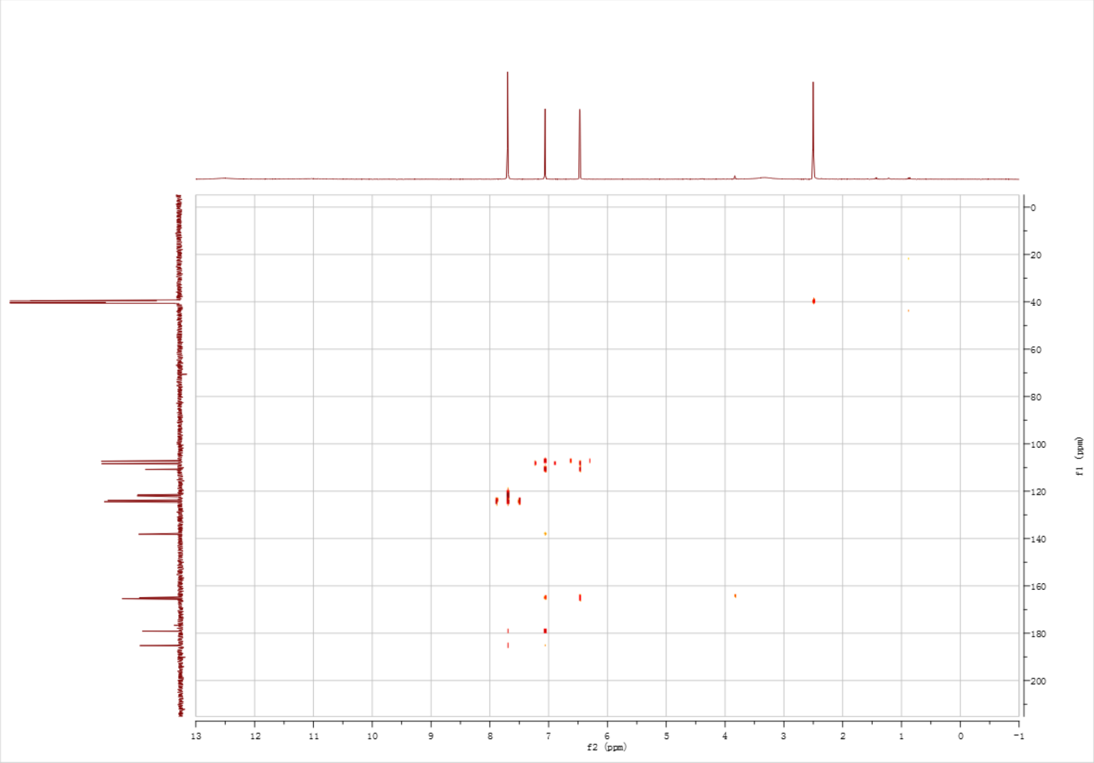

Figure S5. HMBC in DMSO-*d*6 for compound **1**.

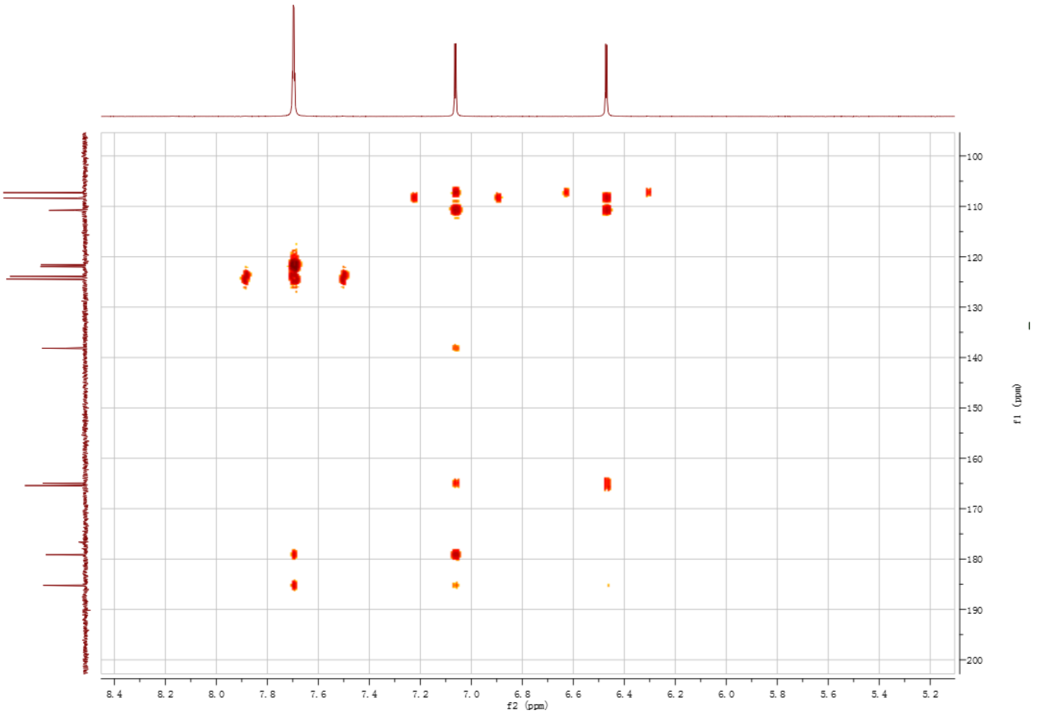

Figure S6. HMBC-2 in DMSO-d_6 for compound **1**.

Acquisition Parameter

Source Type	ESI	Ion Polarity	Positive	Set Nebulizer	0.8 Bar
Focus	Active			Set Dry Heater	200 °C
Scan Begin	50 m/z	Set Capillary	4500 V	Set Dry Gas	5.0 l/min
Scan End	1000 m/z	Set End Plate Offset	-500 V	Set Divert Valve	Waste

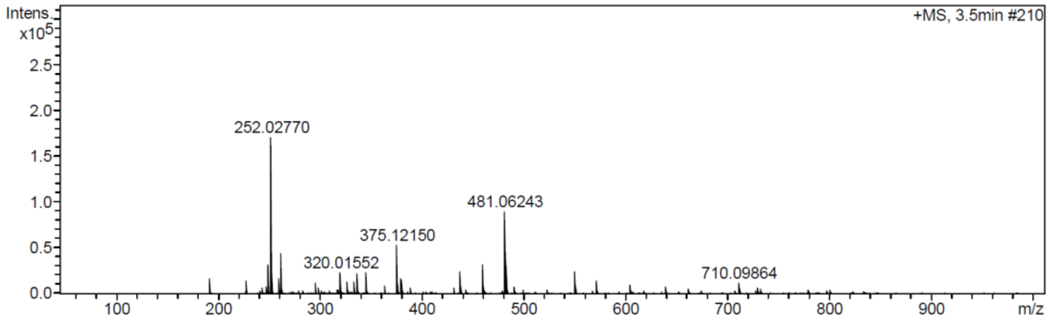

Meas. m/z	#	Formula	err [ppm]	Mean err [ppm]	rdb	mSigma
252.02770						
	1	C 12 H 7 N Na O 4	-3.8	-3.8	9.5	9.51
	2	C 13 H 3 N 5 Na	1.5	1.5	14.5	15.57

Figure S7. HRESIMS for compound **1**.
